# Deficiency of COX7RP, a mitochondrial supercomplex assembly promoting factor, lowers blood glucose level in mice

**DOI:** 10.1038/s41598-017-08081-z

**Published:** 2017-08-08

**Authors:** Sachiko Shiba, Kazuhiro Ikeda, Kuniko Horie-Inoue, Akitoshi Nakayama, Tomoaki Tanaka, Satoshi Inoue

**Affiliations:** 1 Division of Gene Regulation and Signal Transduction, Research Center for Genomic Medicine, Saitama Medical University, Saitama, Japan; 2 Department of Clinical Cell Biology and Medicine, Graduate School of Medicine, Chiba University, Chiba, Japan; 3 Department of Functional Biogerontology, Tokyo Metropolitan Institute of Gerontology, Tokyo, Japan

## Abstract

Mitochondria are essential organelles to efficiently produce ATP by ATP-synthase, which uses a proton-gradient generated by respiratory chain complexes. We previously demonstrated that COX7RP/COX7A2L/SCAF1 is a key molecule that promotes respiratory supercomplex assembly and regulates energy generation. The contribution of COX7RP to metabolic homeostasis, however, remains to be clarified. In the present study, we showed a metabolic phenotype of *Cox7rp* knockout (*Cox7rp*KO) mice, which exhibit lower blood glucose levels after insulin or pyruvate injection. Notably, ATP synthesis rate was reduced in *Cox7rp*KO mice liver, in accordance with decreased percentages of complex III subunit RISP and complex IV subunit COX1 involved in I + III + IV supercomplex fraction. The present findings suggest that COX7RP-mediated mitochondrial respiration plays crucial roles in the regulation of glucose homeostasis and its impairment will lead to the pathophysiology of metabolic states.

## Introduction

Endocrine system plays crucial roles in biological activities and energy homeostasis. People in the developed countries today are prone to have energy excess and reduced physical activities, which often impair the balance of endocrine system and provoke various metabolic disorders including obesity and type 2 diabetes. Energy homeostasis is maintained by various metabolic pathways including glucose and lipid metabolism, which are conducted by multiple endocrine organs^1^. In the context of subcellular functions, mitochondria are important organelles to produce the majority of ATP molecules requiring for cellular functions via ATP-synthase (complex V), which utilizes a proton-gradient generated by respiratory chain complexes (complexes I-IV) involved in their inner membrane.

In mammalian cells, the formation of mitochondrial respiratory supercomplexes or ‘respirasome’ composed of complexes I, III, and IV is considered to facilitate efficient energy generation^2^. We have previously discovered a stabilizing factor for respiratory supercomplex assembly, cytochrome *c* oxidase (COX) subunit 7a-related polypeptide (*COX7RP*)^3^, which was originally identified as an estrogen-inducible gene in breast cancer cells^4^. *COX7RP*, which is also known as *COX7A2L* and *SCAF1*, encodes a 114-amino-acid protein that is structurally similar to a mitochondrial respiratory enzyme COX subunit 7a in complex IV^5^. Gain- and loss-of-function studies of COX7RP showed that the protein is critical for the regulation of muscle activities and the homeostasis of brown adipose tissue (BAT)^3^.

Several groups have also proposed the role of COX7RP in respirasomes. Lapuente-Brun *et al*. independently identified COX7RP as a supercomplex assembly promoting factor (SCAFI)^6, 7^. They found that some mouse strains including C57BL/6 J possess a 6-bp deleted variant of *Cox7rp* gene that encodes a short isoform of COX7RP and C57BL/6 J mice have barely detectable levels of supercomplexes III_2_ + IV and I + III_2_ + IV. Mourier *et al*. also reported that C57BL/6 J as well as C57BL/6 N mice have the short COX7RP isoform, although the mice with the short isoform maintain steady-state levels of complex IV-containing supercomplexes comparable to CD1 strain with long COX7RP isoform^8^. Moreover, recently, Williams *et al*. showed that the C57BL/6 J mice possess the complex IV-containing supercomplex, although there is a tissue variance for the level of supercomplex assembly^9^. Overall, the physiological relevance of COX7RP in supercomplex formation has been widely accepted, yet its precise functions in various tissues remain elusive.

Considering that muscles and BAT are organs responsible for the regulation of insulin sensitivity and energy homeostasis, we next questioned whether COX7RP directly contributes to glucose metabolism *in vivo*. In the present study, we assessed the role of COX7RP in glucose metabolism using *Cox7rp*-knockout (*Cox7rp*KO) mice and revealed that the molecule is a regulatory factor for gluconeogenesis in liver by promoting respiratory supercomplex assembly and ATP synthesis.

## Results

### *Cox7rp*KO mice exhibit altered glucose metabolism

**Figure 1:**
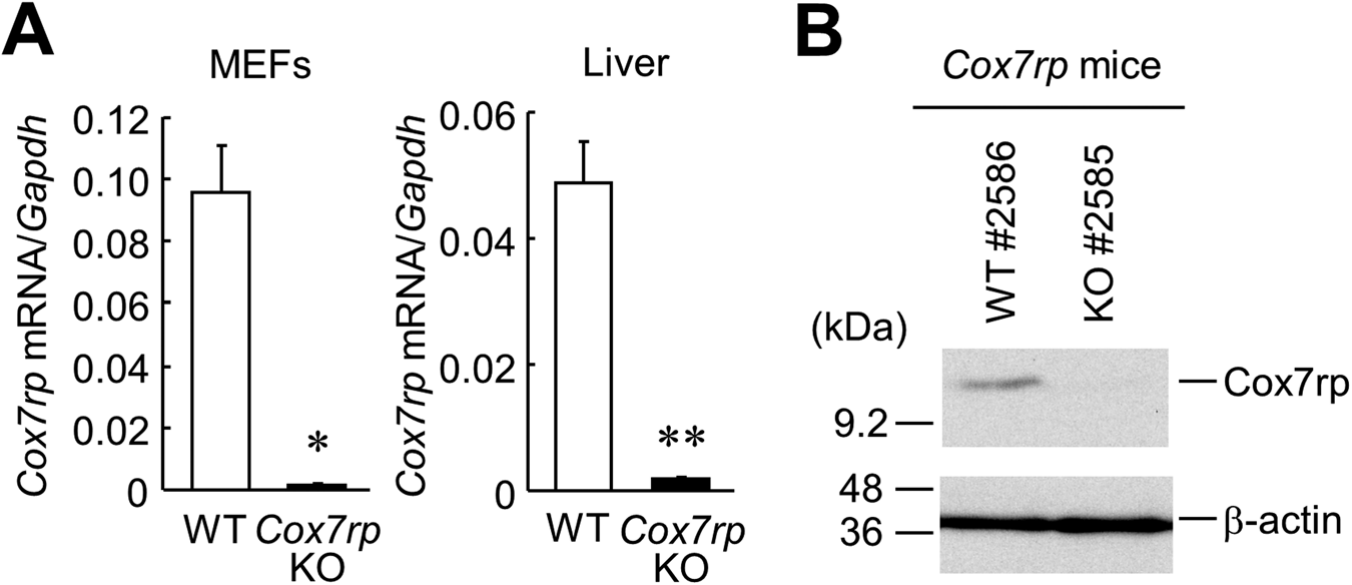
Depletion of *Cox7rp* expression in liver of *Cox7rp*KO mouse. (**A**) Expression level of *Cox7rp* mRNA was quantified by qRT-PCR in MEFs and liver. Data are presented as means ± SEM (*n* = 3). *P < 0.05, **P < 0.01, using Student’s *t* test. (**B**) Western blot analysis of Cox7rp protein expression in liver. Cytosolic fraction was prepared from *Cox7rp*KO and WT livers and subjected to western blot analysis using COX7RP antibody and β-actin antibody for loading control. Unprocessed original scans of the blots are shown in Supplementary Fig. 2.

**Figure 2:**
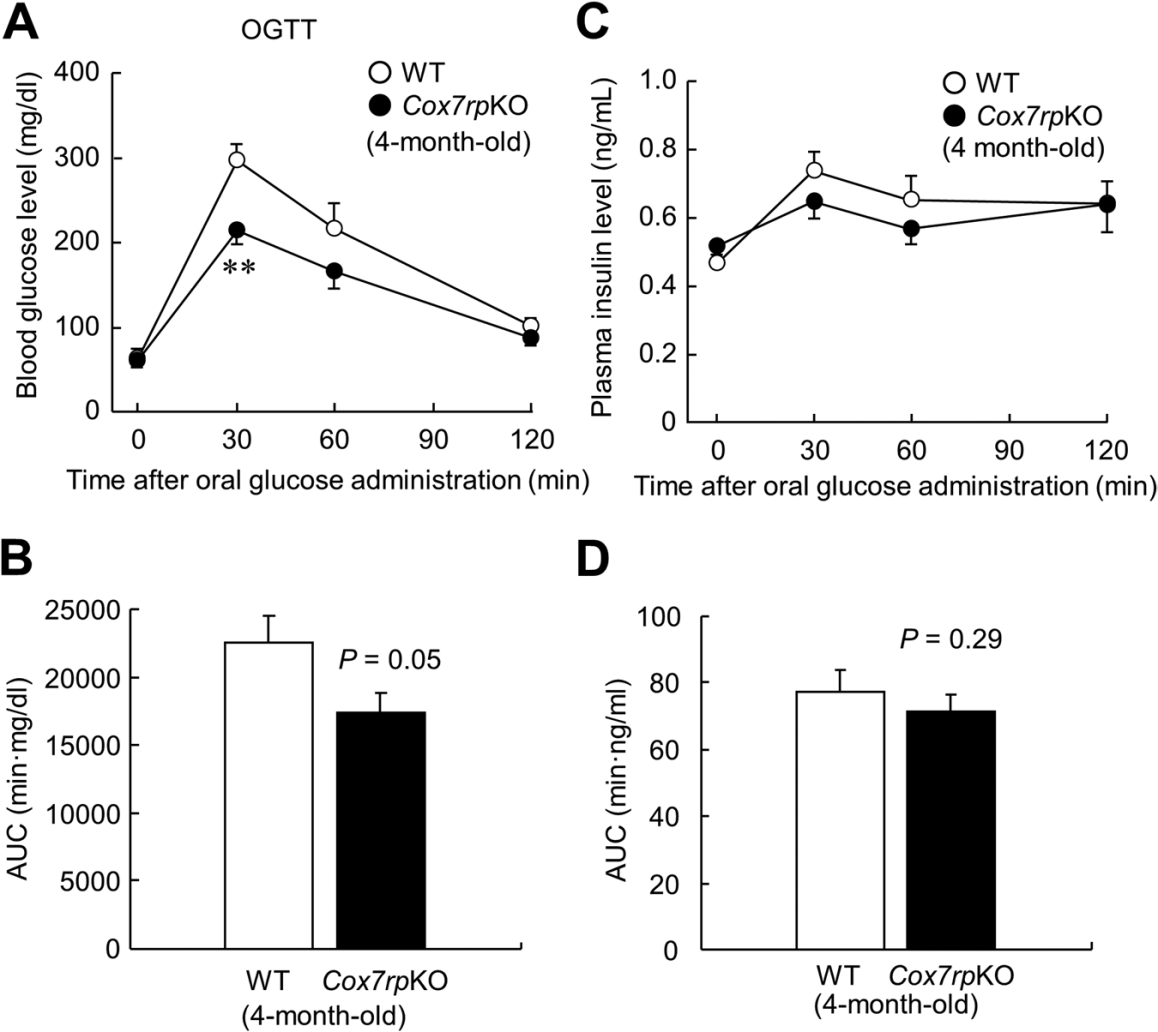
Lower blood glucose levels in 4-month-old *Cox7rp*KO mice *versus* WT mice during oral glucose tolerance test. (**A**) Blood glucose levels during oral glucose tolerance test (OGTT). *Cox7rp*KO and WT mice at 4-month-old were fasted for 16 h, then orally administered glucose (2 g/kg body weight). Blood samples were collected from the saphenous vein at indicated time points after glucose administration. Blood glucose levels were measured using a glucose analyzer. (**B**) Area under the curve (AUC) of blood glucose in OGTT. (**C**) Plasma insulin levels during OGTT. Plasma insulin levels were measured by using ELISA kits. (**D**) AUC of plasma insulin in OGTT. Data are presented as means ± SEM (*n* = 6). *P < 0.05, **P < 0.01, using Student’s *t* test.

**Figure 3:**
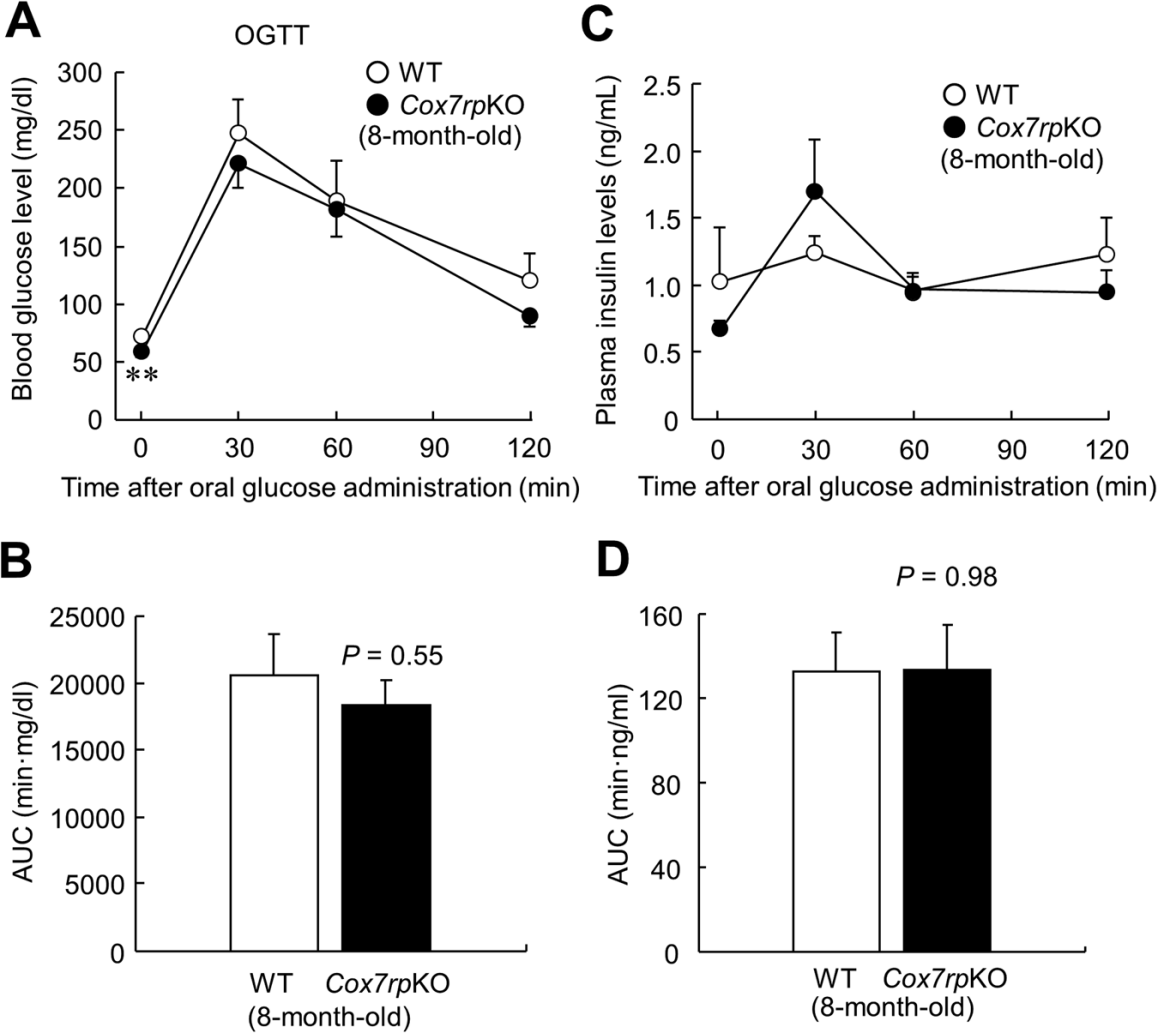
Lower blood glucose levels in 8-month-old *Cox7rp*KO mice *versus* WT mice during OGTT. (**A**) Blood glucose levels during OGTT were examined in 8-month-old *Cox7rp*KO and WT mice as described in Fig. 1. (**B**) AUC of blood glucose in OGTT. (**C**) Plasma insulin levels during OGTT. (**D**) AUC of plasma insulin in OGTT. Data are presented as means ± SEM (*n* = 6). *P < 0.05, **P < 0.01, using Student’s *t* test.

In the previous study, we showed that *Cox7rp*KO mice exhibited a phenotype with reduced muscular activity and decrease in heat production^3^. Here, we questioned whether COX7RP also affects metabolic pathways that closely relate to liver functions because the liver is also one of the essential metabolic organ. To confirm the depletion of COX7RP mRNA and protein in liver of *Cox7rp*KO mice, we performed qRT-PCR and western blot analysis. The *Cox7rp* mRNA expression was scarcely detected in liver of *Cox7rp*KO mice as well as in the mouse embryonic fibroblasts (MEFs) (Fig. 1A). COX7RP protein was expressed in the liver of WT mice but not of *Cox7rp*KO (Fig. 1B). We confirmed that our *Cox7rp*KO mice have a genetic background of 129 strain, which has longer wild-type alleles of *Cox7rp* gene (Supplementary Fig. 1)^6^. The longer *Cox7rp* allele is, however, functionally null and does not produce COX7RP protein. WT littermates that we used have a genetic background of C57BL/6 N, which has shorter alleles of *Cox7rp* gene. To assess a physiological function of COX7RP in glucose metabolism, we performed OGTT in 4-month-old male *Cox7rp*KO and WT mice. OGTT showed that blood glucose levels at 30 min after oral glucose administration were decreased in *Cox7rp*KO mice compared with the same aged WT mice (Fig. 2A). The area under the curve (AUC) analysis for blood glucose during OGTT showed that the AUC values in *Cox7rp*KO mice tended to be lower than those in WT mice, even without a statistical significance (Fig. 2B). Plasma insulin levels during OGTT were not substantially different between *Cox7rp*KO and WT mice (Fig. 2C), thus AUC analysis for plasma insulin showed no significant difference between *Cox7rp*KO and WT mice (Fig. 2D). We performed OGTT also in 8-month-old mice, concerning a possibility that various metabolic pathways may be altered by aging. It is notable that blood glucose level at 0 min was significantly decreased in *Cox7rp*KO mice compared with WT mice (Fig. 3A), although there is no statistical difference in the AUC values between *Cox7rp*KO and WT mice (Fig. 3B). Similar to the results in 4-month-old mice, plasma insulin levels in OGTT were not substantially different between the 2 groups (Fig. 3C) and the AUC analysis also showed no significant difference between *Cox7rp*KO and WT mice at 8 month age (Fig. 3D).

### *Cox7rp*KO mice exhibit lower blood glucose level in insulin tolerance test

**Figure 4:**
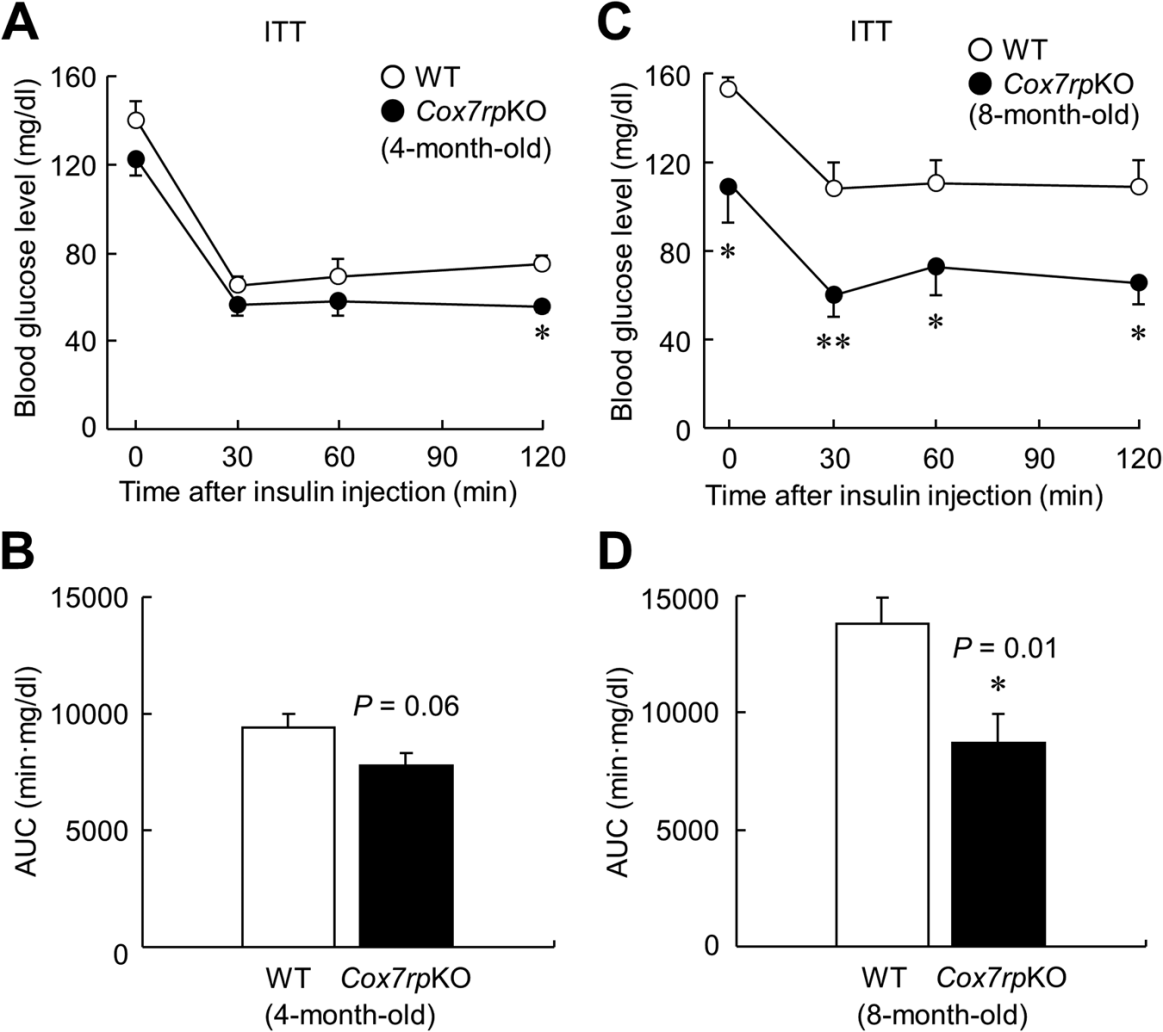
Lower blood glucose levels in 4- and 8-month-old *Cox7rp*KO mice *versus* WT mice during insulin tolerance test (ITT). *Cox7rp*KO and WT mice at 4-month-old and 8-month-old were fasted for 1 h, then injected intraperitoneally with insulin (0.5 U/kg body weight). Blood glucose level (**A**) and the AUC (**B**) in ITT at 4-month-old. Blood glucose level (**C**) and the AUC (**D**) in ITT at 8-month-old. Data are presented as means ± SEM (*n* = 6). *P < 0.05, **P < 0.01, using unpaired Student’s *t* test.

OGTT indicated that *Cox7rp*KO mice have lower blood glucose level without remarkable change of insulin level. To examine the response to insulin, we performed insulin tolerance test (ITT) in *Cox7rp*KO and WT mice. In 4-month-old mice, ITT showed that blood glucose levels at 120 min were lower in *Cox7rp*KO mice compared with WT mice (Fig. 4A). The AUC values in *Cox7rp*KO mice were not significantly different from those in WT mice, nevertheless, there was a tendency that mice exhibited lower glucose levels (Fig. 4B). At 8-month-old, glucose levels during ITT were significantly decreased in *Cox7rp*KO mice compared with WT mice (Fig. 4C). The AUC analysis showed that the glucose levels in 8-month-old *Cox7rp*KO and WT mice were significantly different (Fig. 4D). The data indicate that young *Cox7rp*KO mice have a better ability to maintain circulating glucose levels with a smaller amount of insulin secretion upon glucose load. In 8-month-old, blood glucose levels in *Cox7rp*KO mice were much lower than those in WT mice at all the time-points up to 120 min after insulin stimulation.

### *Cox7rp*KO mice exhibit lower blood glucose level in pyruvate tolerance test

**Figure 5:**
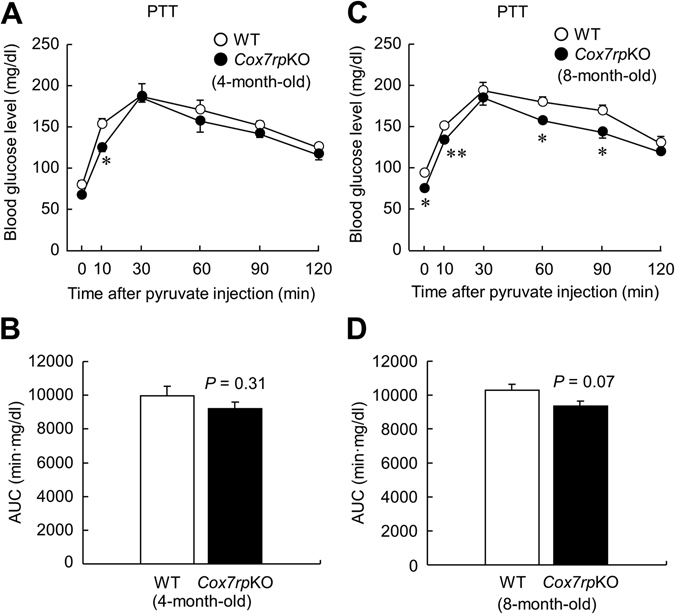
Lower blood glucose levels in 4- and 8-month-old *Cox7rp*KO mice *versus* WT mice during pyruvate tolerance test (PTT). *Cox7rp*KO and WT mice at 4-month-old and 8-month-old were fasted for 16 h, then injected intraperitoneally with pyruvate (2 g/kg body weight). Blood glucose level (**A**) and the AUC (**B**) in PTT at 4-month-old. Blood glucose level (**C**) and the AUC (**D**) in PTT at 8-month-old. Data are presented as means ± SEM (*n* = 4). *P < 0.05, **P < 0.01, using Student’s *t* test.

We next questioned whether the alteration of gluconeogenesis contributes to lower blood glucose levels in *Cox7rp*KO mice. We therefore performed pyruvate tolerance test (PTT) in *Cox7rp*KO and WT mice and found that blood glucose levels were lower at 10 min in 4-month-old *Cox7rp*KO mice after pyruvate injection (Fig. 5A). AUC of blood glucose during PTT showed no significant difference between *Cox7rp*KO and WT mice at 4 month age (Fig. 5B). In addition, blood glucose levels were lower at 0–10 and 60–90 min in 8-month-old *Cox7rp*KO mice after pyruvate injection (Fig. 5C). AUC values of blood glucose for *Cox7rp*KO mice were not statistically different from those for WT mice, yet blood glucose levels tended to be lower in 8-month *Cox7rp*KO compared with WT mice (Fig. 5D).

Taken together, these results show that *Cox7rp*KO mice exhibit a phenotype of reduced blood glucose, which is putatively resulted from the impairment of gluconeogenesis ability or the increase in insulin sensitivity.

### Reduction of respiratory supercomplex assembly in *Cox7rp*KO liver mitochondria

**Figure 6:**
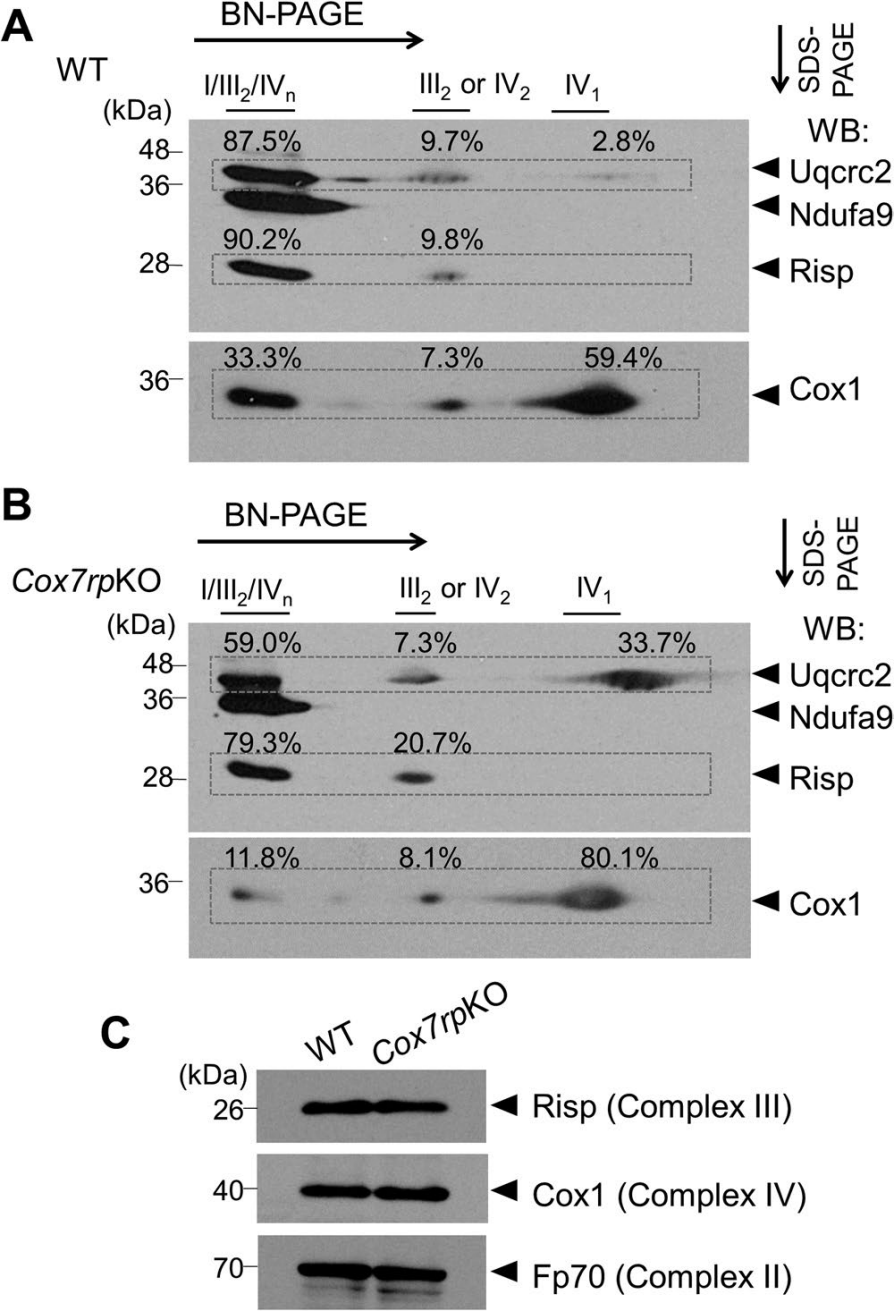
COX7RP promotes assembly of respiratory chain supercomplexes in hepatic mitochondria. Mitochondrial proteins of liver from WT (**A**) and *Cox7rp*KO (**B**) mice at 10-month-old were solubilized with digitonin in concentration of 8 g/g protein, and subjected to BN-PAGE followed by second-dimensional SDS-PAGE. Western blot analysis was performed with antibodies for Ndufa9 of complex I, Uqcrc2 of complex III, and Risp of complex III. The blots were stripped and reprobed with anti-Cox1 antibody. Positions corresponding to the supercomplexes I/III_2_/IV_n_ and complexes III_2_ or IV_2_ are indicated. Percentages of Risp, Uqcrc2, Cox1 signal intensities involved in I/III_2_/IV_n_, III_2_ or IV_2_, and IV_1_ are shown on the images. (**C**) Mitochondrial proteins of liver from WT and *Cox7rp*KO mice at 10-month-old were subjected to SDS-PAGE. Western blot analysis was performed with antibodies for Risp, Cox1, and Fp70 of complex II. Unprocessed original scans of the blots are shown in Supplementary Fig. 2.

**Figure 7:**
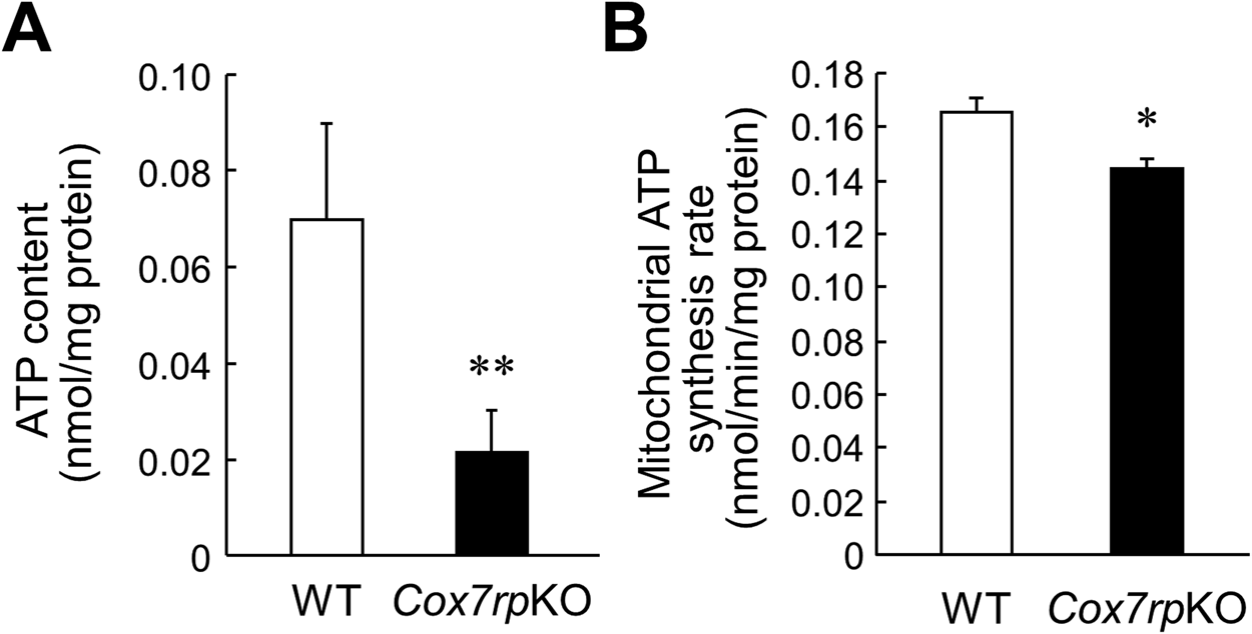
COX7RP deficiency reduces ATP production in hepatic mitochondria. (**A**) Decreased ATP contents in liver of 10-month-old *Cox7rp*KO mice. Data are presented as means ± SEM (*n* = 6). (**B**) ATP synthesis rates in liver of *Cox7rp*KO and WT mice. Data are presented as means ± SEM (*n* = 3). *P < 0.05, **P < 0.01, using Student’s *t* test.

We previously showed that the signal of Rieske iron-sulfur protein (Risp), a complex III subunit and late-stage subunit of supercomplex^10, 11^, was substantially decreased in the assembly of complexes I/III_2_/IV_n_ in digitonin-solubilized mitochondria of *Cox7rp*KO muscle, analyzed by two-dimensional blue native polyacrylamide gel electrophoresis (2D BN-PAGE) with subsequent immunoblotting^3^. Because gluconeogenesis is mainly conducted by liver in mammals, we next investigated whether the extent of respiratory supercomplex assembly in liver mitochondria is related to the gluconeogenesis ability of *Cox7rp*KO mice. Notably, 2D BN-PAGE with subsequent immunoblotting (Fig. 6A,B) revealed that the relative proportion of Risp signal involved in complexes I/III_2_/IV_n_ was lower in *Cox7rp*KO liver (79.3%) compared with WT liver (90.2%). In contrast, the relative proportion of Risp signal into complexes III_2_ was higher in *Cox7rp*KO liver (20.7%) compared with WT liver (9.8%). In regard to Uqcrc2, its incorporation into complexes I/III_2_/IV_n_ seemed to be also lower in *Cox7rp*KO liver (59.0%) compared with WT liver (87.5%). It is also noted that the substantial proportion of Uqcrc2 signal was not incorporated into complexes I/III_2_/IV_n_ or III_2_ but into the far right signal of the 2D gel for *Cox7rp*KO liver (33.7%), in comparison with that for WT liver (2.8%). In terms of complex IV, the proportion of COX1 signal involved in complexes I/III_2_/IV_n_ was also lower in *Cox7rp*KO liver (11.8%) compared with WT liver (33.3%). Thus, the assembly of supercomplex in *Cox7rp*KO liver mitochondria might be reduced compared with that in WT liver mitochondria. In terms of mitochondrial protein loading, amounts of Risp, Cox1 and Fp70 were not substantially different between WT and *Cox7rp*KO livers (Fig. 6C). Consistent with the result of supercomplex assembly, ATP contents in *Cox7rp*KO liver were significantly decreased compared with those in WT mice (Fig. 7A). Moreover, we examined mitochondria-dependent ATP synthesis rates in isolated liver mitochondria from both *Cox7rp*KO and WT mice. The mitochondria-dependent ATP synthesis rate corresponds to per minute produced ATP amount, which is determined by total ATP production subtracted by oligomycin-insensitive ATP production. The results showed that ATP synthesis rate is significantly decreased in *Cox7rp*KO liver mitochondria (Fig. 7B).

Overall, the present data showed that deficiency of COX7RP in mice reduces the relative proportion of mitochondrial supercomplex assembly *versus* individual complexes III or IV and ATP generation in liver, putatively leading to the decrease in blood glucose levels.

## Discussion

The present study shows that COX7RP/COX7A2L/SCAFI plays a critical role in glucose homeostasis, which may be closely associated with its promoting action for respiratory supercomplex assembly that facilitates ATP production in liver mitochondria. We previously discovered that COX7RP is a stabilizing factor for mammalian mitochondrial supercomplex assembly, which contributes to the increase in muscle activity and adaptive thermogenesis *in vivo* ^3^. COX7RP is a nuclear DNA-encoded mitochondrial gene and our gain- and loss-of-function studies of the molecule revealed that it is an essential factor for oxidative phosphorylation.

Jha *et al*. described in their in-gel activity assay that C57BL/6 mice possess COX7RP short form and have only three of the five supercomplexes (SCs), namely, SC 3 (I + III_2_ + IV_2_) and SC 4 (I + III_2_ + IV_3_) are not formed in this strain^12^. They and others also showed the absence of complex III_2_ + IV_1_ in C57BL/6 strain^13^. Cogliali *et al*. also showed the virtual absence of complex III_2_ + IV_1_ and a concomitant reduction in supercomplex I + III_2_ + IV in C57BL/6 liver^7^. We consider that *Cox7rp* deficiency reduces the relative proportion of supercomplex I/III_2_/IV_n_ *versus* complexes III_2_, IV_2_ or IV_1_ as we evaluate by the signal intensity of Uqcrc2, Risp or COX1 in the 2D BN-PAGE. In terms of the incorporation level of Cox1 into supercomplex I/III_2_/IV_n_, we showed that it is not higher in *Cox7rp*KO than WT liver in the Fig. 6. In addition, it is also noted that the substantial proportion of Uqcrc2 signal was detected in the far right band with molecular mass significantly lower than complex III dimer signal. COX7RP has been shown to bind to complex III and stimulate the formation of supercomplex III_2_/IV^7, 13^. We speculate that COX7RP may stabilize complex III formation. It is known that the assembly of supercomplexes is proceeded by gradually collecting individual subunits of complex I, complex III, and complex IV rather than originating from the association of preassembled individual holoenzymes^11^. From that point of view, there is a possibility that a detected signal intensity for a single subunit in BN-PAGE does not correlated to the amount of its cognate respirasome. Thus, the relationship between respirasome abundance and physiological function remains to be clarified. As a future study, *in vitro* import experiments to follow the assembly of subunits of complexes I, IV or III would be useful to assess the assembly of respirasome in *Cox7rp*KO mitochondria.

In this study, blood glucose levels during OGTT were reduced in young *Cox7rp*KO mice whereas no substantial alteration of plasma insulin levels was observed in *Cox7rp*KO mice compared with WT mice. We thus questioned whether insulin sensitivity is enhanced in *Cox7rp*KO mice, although ITT revealed that blood glucose levels in *Cox7rp*KO mice were decreased compared with WT mice and did not recover at the levels of WT mice at 120 min after insulin injection. Intriguingly, blood glucose levels during PTT were reduced in *Cox7rp*KO mice, suggesting that gluconeogenesis in *Cox7rp*KO mice is rather repressed compared with WT mice. It is also noted that 8-month-old but not 4-month-old *Cox7rp*KO mice exhibited significant lower blood glucose levels at 0 min in OGTT compared with WT mice. In addition, the blood glucose levels had a tendency to be lower in 8-month-old *Cox7rp*KO compared with WT mice during the experimental time course in OGTT. The differences of PTT in 8-month *Cox7rp*KO and WT mice will also reveal the contribution of COX7RP in gluconeogenesis, which might be more severely impaired during aging process. Taken together, we consider that the lower blood glucose levels in OGTT at 0 min observed in 8-month-old mice could be explained by impaired gluconeogenesis in *Cox7rp*KO liver. In terms of insulin resistance, plasma insulin levels in 8-month-old *Cox7rp*KO mice were not elevated in OGTT (Fig. 3C), suggesting that the fasting-induced hypoglycemia in *Cox7rp*KO may not be solely explained by the increase in insulin resistance.

Gluconeogenesis is a major pathway of hepatic glucose homeostasis and the primary source for endogenous glucose produced from pyruvate in the fasted state, requiring 6 ATP molecules for a single glucose molecule^14, 15^. In *Cox7rp*KO liver, the impairment of mitochondrial I + III + IV supercomplex assembly would be unfavorable for oxidative phosphorylation, leading to the reduction of total ATP amounts. In the initial step of gluconeogenesis, pyruvate is converted to oxaloacetic acid by consuming ATP. Oxaloacetic acid is converted to phosphoenolpyruvic acid, which is widely utilized as a substrate for multiple metabolic pathways. In terms of the reduced exchange rate of NADH to NAD + , it will weaken the driving force of TCA cycle, which will also result in the reduced uptake of pyruvate into TCA cycle. The altered activity of mitochondrial respiratory chain could also modulate SLC25 carrier protein family^16^, which may also contribute to the reduced uptake of pyruvate into glucose homeostasis. Moreover, AMP level will be increased in contrast to reduced ATP level^17^. High AMP level inhibits fructose 1,6-bisphosphatase activity, which is one of the three rate-limiting enzymes of gluconeogenesis^18^. On the contrary, the elevation of AMP level will activate phosphofructokinase 1, leading to the activation of glycolysis. Taken together, low ATP level in *Cox7rp*KO liver would be involved in decrease of gluconeogenesis. It is because liver is the major tissue that is responsible for gluconeogenesis and this metabolic pathway primarily requires a significant amount of ATP as energy. Alternatively, the improvement of insulin sensitivity may also cause the decrease in blood glucose levels in *Cox7rp*KO mice. It is known that reduced ATP content is anticipated to increase the activity of a fuel-sensing enzyme AMP kinase, which increases insulin sensitivity^19^.

Concerning that the reduction of blood glucose levels after pyruvate load is getting severe along with aging, it could be speculated that other compensatory pathways will be also involved in the glucose homeostasis and aging will modulate the efficacy of those pathways. Lower glucose levels will be also related to the shortage of glycogen or the reduction of fatty acid synthesis, and the energy depletion may further damage mitochondria structure and liver tissues during aging processes. Indeed, we showed that the expression of several mitochondrial carrier proteins are reduced in muscles and BAT of *Cox7rp*KO mice in the previous results of microarray analyses^3^. Thus, it is possible that COX7RP loss could modulate mitochondrial pathways. Overall, we assume that the reduced efficacy of electron transport chain in liver will lead to decrease in ATP amounts and activate catabolic pathways of glucose homeostasis, putatively modulating the levels of various intermediates involved in TCA cycle and associated pathways.

In summary, our results suggest that COX7RP promotes the assembly of respiratory supercomplexes in hepatic mitochondria and increases ATP production, subsequently enhancing gluconeogenesis or affecting insulin sensitivity. COX7RP would be assumed as a novel mediator for glucose and energy homeostasis. Further studies will clarify the precise roles of COX7RP in the regulation of various endocrine metabolic pathways.

## Methods

### *Cox7rp* knockout mice

*Cox7rp*KO mice were generated by Lexicon Genetics using random retroviral gene trapping in 129 strain-derived ES cells as described previously^3, 20^. *Cox7rp*KO mice were born at Mendelian ratios and were fertile. Two pairs of *Cox7rp*KO heterozygous mice were purchased and backcrossed to the C57BL/6 N inbred strain through nine generations to generate *Cox7rp*-deficient mice and WT littermates. For genotyping, genomic DNA derived from tail was used as a template for PCR analysis using specific primers as described previously^3^. To confirm the *Cox7rp* variant alleles coding for short (111 amino acids) and long (113 amino acids) form^6^, PCR was performed using genomic DNA from tails or heart with primers (forward, 5′-CTTTCTTGCTTTGCAGAAGGC-3′; and reverse, 5′-GAAGGCCTCGTTTCAGGTGG-3′). *Cox7rp* PCR products are as follows: long form, 56 bp; short form, 50 bp. All animal experiments were approved by the Animal Care and Use Committee of Saitama Medical University, and conducted in accordance with the Guidelines and Regulations for the Care and Use of Experimental Animals by Saitama Medical University. Mice were maintained in a temperature-controlled room (23 °C) with a 12-h light/dark schedule and fed a standard diet (CE2, CLEA Japan), with free access to water. At 10-month-old, male mice were sacrificed; liver tissues were dissected and immediately stored at −80 °C until analysis.

### Quantitative real-time polymerase chain reaction (qRT-PCR)

Total RNAs were extracted from the mouse embryonic fibroblasts (MEFs) and liver of wild-type (WT) and *Cox7rp*KO mice using ISOGEN reagent (Nippon Gene, Tokyo, Japan). To examine the *Cox7rp* gene expression, quantitative reverse transcriptase-PCR (qRT-PCR) was performed as described previously^21^. Briefly, first strand cDNA generated from total RNA was subjected to qRT-PCR using SYBR green PCR master mix (Applied Biosystems) and the ABI Prism 7000 system (Applied Biosystems). The sequences of PCR primers are as follows: *Cox7rp* forward, 5′-GCAGAAGTTGGCTGGAGCTT-3′; *Cox7rp* reverse, 5′-TATGCTGTCACACTGGAGGTCAG-3′; *Gapdh* forward, 5′-GCATGGCCTTCCGTGTTC-3′; *Gapdh* reverse, 5′-TGTCATCATACTTGGCAGGTTTCT-3′. The comparison of PCR product amounts among differentiation stages was carried out by the comparative cycle threshold (Ct) method, using *Gapdh* as a control.

### Western blot analysis

Cytosolic fraction prepared from liver was resolved using 15% SDS-PAGE, and then electrophoretically transferred onto polyvinylidene difluoride membranes (Millipore). The membranes were probed with anti-COX7RP antibody diluted 1:1,000 (Proteintech), anti-RISP antibody diluted 1:10,000 (Abcam), anti-Cox1 antibody diluted 1:10,000 (Abcam), anti-Fp70 antibody diluted 1:10,000 (Invitrogen) or anti-β-actin antibody diluted 1:5,000 (Sigma-Aldrich). Binding of primary antibodies was detected by horseradish peroxidase-conjugated anti-rabbit or anti-mouse immunoglobulin (Ig)G antibody diluted 1:4,000 (GE Healthcare). Immunoreactive proteins were visualized using enhanced chemiluminescence (Pierce Biotechnology).

### Oral glucose tolerance test (OGTT)

Male *Cox7rp*KO and WT mice at 4- and 8-month-old (*n* = 6 for each group) were fasted for 16 h before OGTT as described^22^. Mice were challenged with orally administered D-( + )-glucose (2 g/kg body weight). Blood samples were collected from the saphenous vein at 0, 30, 60, and 120 min after glucose administration. Blood glucose levels were measured using a glucose analyzer (Sanwa Kagaku Kenkyusho Co., Ltd.). Plasma insulin levels were measured using the Mouse Insulin ELISA KIT (S-type) (Shibayagi Co., Ltd.).

### Insulin tolerance test (ITT)

Male *Cox7rp*KO and WT mice at 4- and 8-month-old (*n* = 6 for each group) were fasted for 1 h before ITT as described^22^. Mice were injected intraperitoneally with insulin (0.5 U/kg body weight). Blood samples were collected from the saphenous vein at 0, 30, 60, and 120 min after injection of insulin. Blood glucose levels were measured using a glucose analyzer (Sanwa Kagaku Kenkyusho Co., Ltd.).

### Pyruvate tolerance test (PTT)

Male *Cox7rp*KO and WT mice at 4- and 8-month-old (*n* = 4 for each group) were fasted for 16 h before PTT. Mice were injected intraperitoneally with sodium pyruvate (2 g/kg body weight). Blood samples were collected from the saphenous vein at 0, 10, 30, 60, 90 and 120 min after injection of pyruvate.

### Calculation of glucose and insulin area under the curve (AUC)

The areas under the blood glucose level *versus* time curve of OGTT, ITT, and PTT, and the plasma insulin level *versus* time curve of OGTT were calculated for each subject in *Cox7rp*KO and WT mice at 4- and 8-month-old.

### Measurement of ATP

ATP concentration in liver was quantified using tissues obtained from 10-month-old of *Cox7rp*KO and WT mice. Frozen liver samples were homogenized in 1.0 mL of ice-cold 10 mM HEPES-NaOH (pH 7.4) and 0.25 M sucrose buffer. Supernatants were centrifuged at 1,000 *g* for 10 minutes at 4 °C. One hundred μL of supernatant was pipetted into each well of a black non-phosphorescent microplate, placed in a MicroLumat Plus luminometer (Berthold Technologies), and processed by the addition of 100 μL of ATP luminescent reagent (TOYO B-Net Co.,Ltd.). ATP concentrations were calculated from a calibration curve constructed by the simultaneous measurement of standard ATP for each experiment. ATP synthesis in mitochondria was measured as described previously^3^.

### Blue native-polyacrylamide gel electrophoresis (BN-PAGE)

Liver tissues were homogenized with a glass-teflon homogenizer in a buffer containing 10 mM HEPES-KOH (pH 7.4), 0.22 M mannitol, 0.07 M sucrose and 0.1 mM EDTA as described^3^. The liver extracts were centrifuged at 500 *g* and the supernatants were further centrifuged at 10,000 *g* to precipitate mitochondrial fraction. The mitochondrial fraction (100 μg protein) was suspended in 15 μL of a buffer containing 30 mM HEPES-KOH (pH 7.4), 150 mM potassium acetate and 10% glycerol. Digitonin (digitonin/protein ratio 8 g/g) was added to solubilize the mitochondria. After 30-min incubation at 4 °C, solubilized proteins were obtained as supernatant fraction by centrifugation at 22,000 *g*. Solubilized proteins were supplemented with 0.5 μL of sample buffer (5% coomassie brilliant blue G-250 in 1 M 6-aminocapronic acid). Stacking (4%) and separating gels with stepwise 8, 9, 10 and 11% were cast and electrophoresed according to the method of Scägger and von Jagow^23^. Second-dimensional SDS-PAGE and immunoblotting were performed according to standard protocols, and blotted membranes were probed with anti-NDUFA9 (Invitrogen), anti-UQCRC2 (Abcam), anti-RISP (Abcam) and anti-COX1 (Abcam).

### Statistical analysis

Significance of differences between two groups was analyzed by unpaired Student’s *t* test.

## Electronic supplementary material

Supplementary info
