# Supplementary material for: Deficiency of COX7RP, a mitochondrial supercomplex assembly promoting factor, lowers blood glucose level in mice
Source: Sci Rep. 2017 Aug 8;7:7606. doi: 10.1038/s41598-017-08081-z (PMC5548899; doi:10.1038/s41598-017-08081-z)
Supplement: Supplementary file 1 — Supplementary info [file 41598_2017_8081_MOESM1_ESM.pdf]

Supplementary Information

Deficiency of COX7RP, a mitochondrial supercomplex assembly promoting factor, lowers blood glucose level in mice

Sachiko Shiba[1], Kazuhiro Ikeda[1], Kuniko Horie-Inoue[1], Akitoshi Nakayama[2], Tomoaki Tanaka[2] & Satoshi Inoue[1,3,*]

[1]Division of Gene Regulation and Signal Transduction, Research Center for Genomic Medicine, Saitama Medical University, Saitama, Japan.

[2]Department of Clinical Cell Biology and Medicine, Graduate School of Medicine, Chiba University, Chiba, Japan.

[3]Department of Functional Biogerontology, Tokyo Metropolitan Institute of Gerontology, Tokyo, Japan.

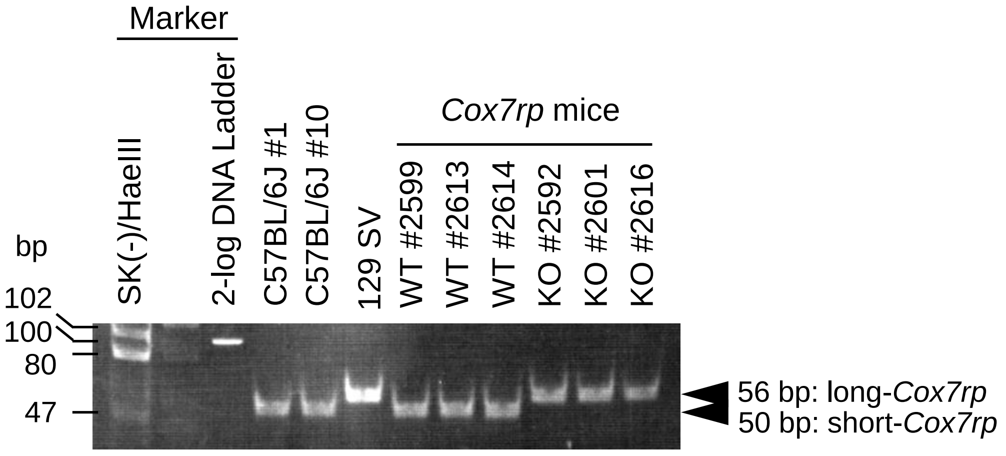

Supplementary Figure 1. PCR analysis for genotyping of long- (113 amino acids) and short- (111 amino acids) form *Cox7rp* alleles. PCR was performed with specific primers using genomic DNA from heart (129 SV) or tails (others). PCR products are as follows: long form (129 SV mouse and *Cox7rp*KO mice), 56 bp; short form (C57BL/6J and WT mice), 50 bp.

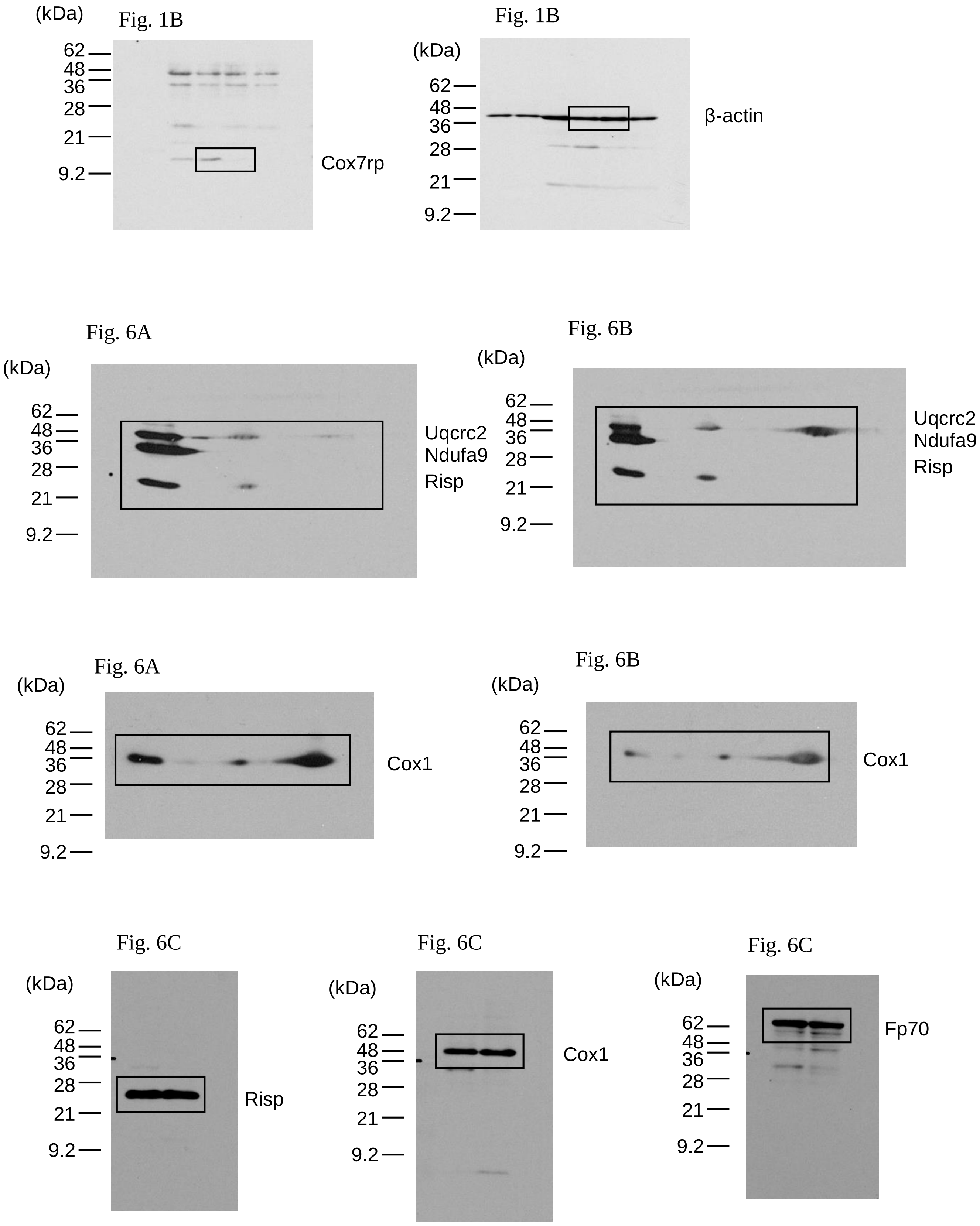

Supplementary Figure 2. Unprocessed original scans of the blots.
